# Cleft Team Clinicians’ Perspectives on the Process of Patient Transition from Childhood to Adulthood in New Zealand

**DOI:** 10.1177/10556656241299910

**Published:** 2024-12-05

**Authors:** Maia Rose Entwistle, Phoebe Macrae, Kenny Ardouin

**Affiliations:** 1 School of Psychology, Speech and Hearing, 2496 University of Canterbury, Christchurch, New Zealand

**Keywords:** transition of care, quality of life, team care, psychosocial adjustment, speech therapy

## Abstract

**Objective:**

This study sought to understand cleft team clinician experiences of transitioning patients from pediatric to adult cleft care services in New Zealand.

**Design:**

The qualitative study conducted 4 interviews and 4 focus groups with Cleft Team clinicians either in person or over Zoom. Data were analyzed using inductive thematic analysis to identify themes.

**Participants:**

Twelve Cleft Team clinicians from 4 Health New Zealand cleft services.

**Results:**

Patients are treated by the same clinicians both before and after transition, however, changes at age 16 include gaining treatment decision-making autonomy, inpatient stays within general plastic surgery wards, and evolving life experiences outside of the hospital. Clinicians reported unmet psychosocial needs of patients and their families, and difficulties experienced by patients returning to cleft services as adults. The development of peer support networks between adolescents and young adults who have experiences of cleft care is also recommended.

**Conclusions:**

Ongoing cleft care provision and expansion of services are recommended. Furthermore, future studies to understand patients’ perspectives of transition are paramount, and incorporating patient voice into any proposed interventions is essential. Potential supports for families of patients with a cleft should also be further investigated, and the application of international standards would promote increased consistency and collaboration between the practice of Cleft Team clinicians around the world.

## Introduction

In most services within the New Zealand healthcare system, including cleft care, patients transition from pediatric to adult services at the age of 16.^1^ Several processes and recommendations are documented within the literature to facilitate patients’ transition from pediatric to adult cleft care at the age of 16 years old (transition).^2–9^ These recommendations include assessing the concerns of patients and their families and counseling them, and supporting patients to engage in their treatment decision-making and take responsibility for their health.^10^ In 2023, the Cleft Lip and Palate Association provided up-to-date protocols within the United Kingdom, including providing support networks to families, and clinical psychology services for patients of all ages.^11^ The United States^12^ and Australia^13^ employ similar protocols which address the holistic well-being of patients and their families throughout this time. However, some countries are yet to establish formal cleft care pathways that address transition to adulthood for patients.^14^

As is the case in many jurisdictions, under New Zealand law, when patients turn 16, they gain autonomy over their treatment decision-making.^15^ Within cleft care, it is around this age that people with cleft often develop increasingly negative perceptions of the hospital setting and encounter anxiety surrounding clinical decision-making.^5,16,17^ Furthermore, the likelihood of adolescents experiencing social isolation from their peers is increased as they undergo numerous hours of treatment,^7,16^ while their peers encounter new experiences outside of the hospital.^16^ These challenges collectively increase patients’ risks of developing various psychological concerns, including depression, anxiety, and treatment-related fatigue.^3–5,7,8,17,18^

The implementation of psychological screening and monitoring protocols^6,7,10,19^ and inclusion of Cleft Team Clinical Psychologists within cleft units has been previously recommended to support patients’ clinical decision-making, relationship-building, and social confidence throughout their treatment journey,^3,10,14,16,17^ and to mitigate anxieties associated with treatment.^16^ However, routine psychological screening and Clinical Psychology provision within cleft services remains limited, often non-existent in many countries.^20^ Despite the lack of Clinical Psychology services, Cleft Team clinicians from all disciplines are encouraged to be attentive to patients’ emotional well-being and engage in frequent communication with them about their whole of life.^8,10,16,18^

Although patients’ experiences of transition are understood to some degree in other countries,^2,8,16,21^ the perspectives of patients in New Zealand towards their transition are minimally understood,^20^ and clinicians’ perspectives have not yet been explored. Clinicians have significant involvement and influence in each patient's cleft treatment journey and play a role in supporting the positive psychosocial adjustment of patients with cleft and their families.^10^ This study aims to identify clinicians’ experiences of supporting patients and their families, including any practices they utilize to aid transition, and clinician perspectives of any concerns that arise for patients during this time.

## Methods

### Study Design

Ethical approval for the study was sought and granted by the University of Canterbury Human Research Ethics Committee. The research consisted of a semi-structured qualitative interview design study. The interview schedule is outlined in Table 1.

**Table 1.** Interview Schedule.

Prompt questions
1. What is the process that you/your Cleft Team follow(s) to transition patients to adult care?
2. Do you feel that transition from pediatric to adult care is currently managed effectively within your service?
3. What are some of the key changes within your service that occur when someone reaches the age of 16? (eg, any changes to wards, treatment protocols, treating/post-surgical teams, etc.).
4. In your experience, how do parents/caregivers/families/whānau manage the delegation of decision-making from caregivers to the patient?
5. What (if any) supports are provided to help patients and their whānau [family] to manage this transition?
6. In your experience and observations, what are some of the concerns/issues that patients and whānau present with around the time of transition?
7. What do you feel are some of the facilitators and/or barriers that help or hinder a smooth transition for a patient and their whānau?
8. What resources/materials could be useful to help patients and their whānau adjust to adult cleft care?
9. What role do you see Cleft New Zealand having to support the transition from pediatric to adult cleft services?
10. When supporting people to prepare for adulthood, what do you feel are important aspects to provide patients and their whānau support with?

### Recruitment

Eligible participants were clinicians employed by 1 of the 5 Health New Zealand cleft services within New Zealand. Eligible Cleft Team clinicians included Speech Language Therapists, Plastic Surgeons, Orthodontists, Cleft Team Dentists, Clinical Nurse Specialists/Cleft Co-ordinators, and Clinical Psychologists (where available). These clinicians were selected due to their involvement with patients affected by cleft throughout the transition period, from pediatric to adult care. Clinicians were invited via email to participate in the study and to indicate their availability and preference for an interview or focus group.

### Data Collection

Sessions were facilitated by the lead researcher during one-to-one interviews, or within focus groups alongside other colleagues within their Cleft Team. Sessions were up to 60 minutes in duration to cater for clinicians’ schedules. Interviews and focus groups took place between April and May 2023, either in person or over Zoom, depending upon the preference and geographical locations of participants. All meetings were audio recorded using a digital voice recorder and subsequently transcribed with the assistance of Otter.ai Transcription Software. Zoom software was also used to record online meetings.

Prior to the interview or focus group, participants were provided an overview document describing the aims of the study and were prompted to consider and reflect on their experiences supporting patients and their families throughout transitional periods, any practices they utilize to aid transition, and potential concerns that arise for patients during this time. Participants were reminded to maintain patient confidentiality, and that although questions would be provided to prompt discussion, they were free to discuss pertinent perspectives and information as they desired.

### Data Analysis

Using Otter.ai Transcription Software, interviews were transcribed verbatim. The lead researcher reviewed and edited the AI-generated transcriptions to ensure these were consistent with the audio recordings. Pseudonyms were generated to replace identifiable information, to maintain the confidentiality of participants, and the confidentiality of their colleagues, patients, and associated family members discussed.

The dataset was analyzed qualitatively using inductive thematic analysis.^22^ Inductive thematic analysis is recommended when completing a scoping study with few participants where little existing information is known. It allows for a data-driven approach where the resultant coding and themes are derived from the data without preconceived codes or themes. This research method operates under the premise that the interview questions are prompts to elicit broad discussion on the topic, rather than a series of specific questions for which researchers are seeking direct answers that are often quantifiable. Transcriptions were analyzed and quotes of interest were identified. Transcriptions were independently analyzed by 2 researchers for interrater reliability. Discrepancies were discussed with a third researcher until consensus was reached. Quotes were then coded, with similar quotes ascribed the same code. Once initial coding was complete, codes were reviewed and refined by the research team. Similar codes were grouped together to form themes and subthemes.^22^

## Results

### Participants

Twelve respondents from 4 cleft units from across New Zealand chose to participate in the study. The participants included six speech-language therapists, 2 Cleft Clinical Nurse Specialists, 2 plastic surgeons, 1 orthodontist, and 1 clinical psychologist.

### Thematic Analysis

Four individual interviews and 4 focus groups were conducted. Four themes and their associated subthemes were identified using inductive thematic analysis. These themes indicate the topics which participants most commonly chose to discuss. Namely, these 4 themes are (1) overview of current cleft services at age 16 and beyond, (2) overview of patient and family transitions into adulthood, (3) the value and role of Cleft Team Clinical Psychologists, (4) the need for adolescent-specific resources and services.

Themes and subthemes are presented in detail in Table 2 including summaries and exemplar participant quotes. Shortened quotations include (…), and additions represented by [xxx] for clarification. Participants collectively referred to as “all” include all or all but one, “most” include more than half, and “some” include less than half, but more than 2 as per qualitative data reporting conventions described by Hill et al.^15^

**Table 2.** Inductive Thematic Analysis Results

*Theme 1: Current cleft services at age 16 and beyond*		
*Subtheme*	*Summary*	*Exemplar quote(s)*
Cleft teams wish to remain as 1 service across the lifespan	Most clinicians considered having one service across the lifespan was considered advantageous for maintaining clinician-patient communication and relationships.	“We’re not really aware of it [age 16] being a transitional time. So, from the patient's point of view, there are things that are different, but from a service point of view we just continue doing what we do … presumably, it has an impact on the person.” (Participant 4)
Discrepancies in care between cleft teams and other services	Some clinicians commented on differing practice between cleft teams.	“The different [cleft] teams in New Zealand are actually offering different services … [what you’re entitled to] depends [on] what [sic] city you live in.” (Participant 1)
*Theme 2: Considerations for patient and family transitions into adulthood*		
*Subtheme*	*Summary*	*Exemplar quote(s)*
Every patient's transition is unique	Most clinicians detailed variabilities in patients’ experiences of transition and ability to cope with associated changes. Patients who demonstrated resilience and had strong familial supports were considered to manage transition more smoothly.	“One adult, or young adult who I've had experience working with, I think she's had a reasonably positive transition … just depends on the person. She's got good [familial] supports around her as well.” (Participant 8)
Adolescence is a time of significant change	Most clinicians acknowledged the age of 16 as a period of holistic transition for patients with a cleft. Most felt that patients exhibit increased readiness to take responsibility for their clinical decision-making at 16, however, their commitments to non-medical priorities influence this.	“When their [parents’] kids are getting to that age, there's lots of other life transitions they are naturally navigating anyway, that when it comes time to a medical appointment, it just makes sense that okay, well, this is changing as well.” (Participant 2) “A lot of them [patients] will just say, I don't want it, I'm not ready now … I've just finished schooling and I'm going on an OE … Or, I've gone to university.” (Participant 7)
Respecting patient autonomy from an early age	All clinicians reported striving to respect children's decision-making autonomy to increase their treatment cooperation and improve their treatment experiences and outcomes. All participants reported seeking assent as soon as patients are old enough to understand their options. Additionally, some participants commented on their tendencies to request to meet with parents and patients separately to understand their individual preferences.	“They’re technically still under the care of their guardian, or their family or their parent, they're not able to make their own independent decisions yet … we don't want them to come in here and be forced into doing stuff and hate the hospital.” (Participant 10)
*Theme 3: The need for cleft-related psychological services*		
*Subtheme*	*Summary*	*Exemplar quote(s)*
Psychological aspects of cleft care are not adequately resourced	Many clinicians reported that patients’ physical presentation are often prioritized over their psychological well-being. Some reported that they desire that patients’ holistic priorities and concerns be addressed, however, due to resource constraints, patients have limited consultation time with clinicians to address these, and their surgical need (or lack thereof) dictates their discharge.	“You [patient] come in, you've got ten minutes to see every single person in the team and get something from it. That's not going to benefit the patient. That's just for the team to put a tick in their box and say does this person need more surgery? Yes, no. Do they need speech therapy? Yes, no, goodbye.” (Participant 6)
Psychosocial distress often goes undetected	Some clinicians detailed occasions of their patients conveying psychological distress, and most reported that psychological distress is potentially going undetected, unreported, or being downplayed. Reasons include a lack of psychology clinicians to support patients’ psychological well-being, or not having clear pathways to support those who have disclosed psychological distress to clinicians. The implementation of ‘Patient Reported Outcome Measures’ (PROMS) was discussed by one participant, a tool designed to support clinicians’ identification of patients’ psychological well-being concerns.	“They [patients] could come into a clinic room seeing, you know, surgeon, the SLT. They seem okay, but I've looked at their screener and kind of gone, well there's a couple of things you've circled on here that I might want to talk to you about, and then you talk to them separately and it's just like these huge amounts of distress, and you wouldn't pick it while they're sitting in front of the MDT [multidisciplinary team].” (Participant 2) “Nationally, we're going to start using PROMS, which is patient-reported outcomes … that's going to give us a really good idea of what's on top for them.” (Participant 6)
Managing parental input beyond the age of 16	Some clinicians reported patients often continue to seek their parents’ support with clinical decision-making after transition. However, adolescent patients can have differing treatment desires from their parents and some clinicians suggest that supports be implemented to improve families’ management of this change.	“Very few sixteen-year-olds of legal consent age suddenly say, no, actually, this is my speech issue. This is my breathing issue.” (Participant 9) “Some parents want minor adjustments performed to chase perfection but often the patient wants to be left alone, requesting that it's their life/body. The converse also occurs, where an adolescent may have become fixated on one thing that is difficult to improve surgically.” (Participant 10)
Patients and families need support to adjust to having a cleft	Most participants believed patients’ and families’ perceptions towards cleft and cleft care to generally be negative. This was noted particularly for families with international backgrounds who often had higher expectations for New Zealand's cleft services than those that are available.	“There are very high expectations in other countries, and so when you come to New Zealand, and you receive the experience you receive here in the Cleft Team, I think that it will fall quite far short of expectations.” (Participant 6) “The family has such a powerful role to play in how these children grow up and see themselves. And so I think if you're looking for resources, creating resources [designed to support families’ positive perceptions of cleft] in my mind, that would be an enormous area to help families and patients.” (Participant 9)
A need for Cleft Team Clinical Psychologists	All clinicians reported feeling that psychological services form an essential part of the cleft service, despite this not being generally available in New Zealand.	“I think that that's where psych support should be accessible [transition] … I think it's a critical age, the young people, when they're making that transition into what they are, young adults … having a psychological support is probably the biggest thing, and the barriers here are that that is not readily available.” (Participant 6)
*Theme 4: The need for adolescent-specific resources and services*		
*Subtheme*	*Summary*	*Exemplar quote(s)*
Difficulties maintaining contact with patients	Some clinicians who coordinate appointments reported that maintaining contact with patients after their transition was often challenging as patients left the family home and have not updated their contact details with the cleft team.	“There's some families who may not have access to technology … there's also the thing of just remembering I guess, with those areas, you know, literacy and people's levels of literacy, which could be limited.” (Participant 6) “If I'm calling somebody in those early twenties, yeah, I call their mum because it's the number that we've got on record, but I do normally start it by you know, introduce myself and say, hey, I'm wanting to book such and such for cleft clinic, should I be talking to you? Or do you want me to talk to such and such?” (Participant 7)
Open communication regarding treatment decisions	Most clinicians reported that patients typically have multiple opportunities to engage in clinical consultations before committing to a procedure, and all clinicians reported a preference to delay elective cleft procedures until patients themselves are motivated for them. Clinicians reported that patients are also encouraged to frequently discuss their options and preferences with their families.	“Where there is uncertainty or inconsistency between patients and their families, and the procedure in question is non-urgent, we offer more consultations until we all feel convinced that this is the way forward. This usually results in success for the whole family unit.” (Participant 10)
The necessity for increased supports and resources for patients	Some clinicians discussed how a patient's understanding of cleft and cleft treatment is a facilitator to their transition. Most clinicians reported a need for increased support and resources for patients throughout their transition and the potential for support groups to arrange peer support opportunities for patients throughout, and after their transition.	“Poor understanding is a barrier, good understanding is a facilitator. … they [patients] have generally a reasonable understanding anyway, but some kids don't … I would imagine that the kids that have sort of grown up with a better understanding of cleft might have an easier time taking a bit more ownership over that decision-making.” (Participant 2) “If Cleft New Zealand would take on the role of identifying people who wanted a mentor and people that would be a mentor. That would seem brilliant.” (Participant 9)
Increasing opportunities for patients to connect	Some clinicians reported that resources including role models, peer support and online networks may be beneficial for patients from the age of 16 to enhance their management of transition.	“If there was a network of young people with cleft, who were able to talk to each other … if someone's thinking about a procedure, and they can ask, you know, has anyone had this? … I've got the medical information from the team, but I want to know, the experience of somebody who's had this.” (Participant 2)
Portrayal of transition	Most clinicians desired for patients to receive more information regarding their potential transition experiences, reporting that information shared is generally procedure-related. One clinician discussed that care must be taken with the implementation of additional resources throughout this time to ensure transition is not conveyed negatively to patients, and another felt that creating additional resources focussed on transition would be unnecessary.	“One time I was working with somebody who was like, I know all the information, but I really want to know what the experience was like … she did reach out to Cleft New Zealand and heard back but it was kind of directing her to go back to her team and ask questions, and it just wasn't quite what she was after.” (Participant 2) “… [transition focussed processes and resources were] appropriate for the diabetics, the rheumatic fever children and the respiratory children, absolutely, but it really didn't fit in with the surgical component … it just wasn't relevant.” (Participant 5) “We [clinicians] don't see that as a big transition, but maybe they [patients] do see that as a big transition … so I wouldn't want them thinking, oh at sixteen, everything's going to stop and everything's going to change. So we don't want them to anticipate that there's going to be a problem.” (Participant 4)

In summary, clinicians were clear that patients are cared for by the same team of clinicians both before and after transition. As a patient transitions from pediatric to adult cleft care, they gain treatment decision-making autonomy, and experience inpatient stays within general plastic surgery wards compared to pediatric wards, and evolving holistic life experiences. Clinicians have detailed their observations of the significant impact these experiences can have on patients and their families. Resultantly, several recommendations were expressed by clinicians to support patients’ transition. These include the development of resources and programs to support patient understanding of cleft and its treatment, and opportunities for patients to access peer support. The current lack of psychological support for patients and their families, and clinicians’ desires for increased consistency and collaboration between each cleft team's approach to transition were also discussed.

## Discussion

Cleft care within New Zealand has traditionally been derived from best practice models within the United Kingdom^20^; however, participants who have experienced working in other countries acknowledged that New Zealand Cleft Teams’ single-service approach to transition differentiates it from some international services (including those of the modern-day United Kingdom). Although there is no single approach to managing transition within cleft care globally, clinicians further report that families traveling from other countries tend to have higher expectations of New Zealand cleft care than those available. For example, in contrast to some other locations, patients are unable to choose from a selection of treatment providers, undergo elective surgical procedures such as rhinoplasty with minimal waiting times, or continue to access cleft-related treatment at any age. Clinicians reported that the age of 16 is not formally recognized as a time of transition for patients with a cleft, and that although existing treatment plans are realized beyond this age, there is no formal pathway for people with cleft to return for additional care following their discharge in early adulthood.^23^ Therefore, countries such as New Zealand should take steps to ensure patients have access to quality services aligned with international best-practice standards.

In countries where transition from pediatric care is formally recognized as a part of the cleft treatment journey, specific protocols are implemented to ensure patients are well supported. For example, in the United Kingdom,^11,24,25^ the United States,^12^ and Australia,^13^ published standards exist which are intended to be followed by all Cleft Teams. Unfortunately, many countries do not have formal service specifications for cleft, resulting in discrepancies between Cleft Teams’ approaches towards transition, and other cleft services; discrepancies which were reported by clinicians within this study. Clinicians reported inadequacies in funding and clinical resources to be barriers to standardization of cleft care, a finding echoed by previous research which demonstrated several clinical outcomes for people in cleft to be poorer than in some other parts of the world.^20^ Cleft Teams have conveyed openness to managing transition differently throughout interviews and focus group discussions, and some detailed New Zealand's existing efforts to increase collaboration between Cleft Teams to provide greater consistency nationally. However, the varying opinions towards current practice in New Zealand and its potential for improvement detailed by participants throughout this study indicate that formal protocols similar to those used internationally should be implemented to ensure patients’ successful transition to adulthood. It is suggested that such initiatives be developed in conjunction with patient and public involvement groups to ensure patient voice is central to such proposals.

Participants reported that patients’ experiences of transition and its effects on their development and well-being vary depending on their treatment intensity, external supports, lifestyles, and relationships, a finding that has been corroborated by previous research.^16^ Furthermore, participants reported several clinical changes encountered by patients at the age of 16 which are also detailed in previous studies. These include gaining treatment autonomy,^16^ inpatient stays within general plastic surgery wards,^8^ and having additional surgical opportunities.^18^ To support patients’ independent treatment decision-making at 16, participants reportedly encourage young patients to participate in their clinical consultations and decision-making before transition,^7,10^ and discuss their concerns with clinicians and family members,^4,5,7,16^ a well-utilized and well-evidenced practice.

Participants also outlined holistic changes patients encounter outside of the hospital environment, including forming new friendships and romantic relationships, and completing secondary/high school studies.^8,17,26^ Some discussed the impact that these non-medical commitments can have on patients’ clinical decision-making, reporting that treatment plans may interfere with significant events in patients’ lives such as beginning new careers, or starting their own families.^7,10^ Therefore, patients’ holistic life experiences, goals, and commitments should be factored into their clinical consultations and decision-making with clinicians. Participants report that incorporating patient-reported outcome measures into New Zealand cleft services aims to support clinicians’ identification of patients’ perspectives and concerns towards their cleft and treatment.

There are consistent reports that patients with a cleft are at a greater risk of developing psychological problems than the general population,^3–8,18,26^ and some participants discussed their efforts to ask patients more questions about their psychological wellbeing. Several participants reported that patients’ psychological distress may often remain undetected and clinicians do not feel equipped to appropriately address patients’ disclosures of distress. To sufficiently investigate and address patients’ psychological well-being in the longer term, Cleft Team Clinical Psychologists should feature on cleft teams as standard^8,14,16,18,19^ and utilize screening tools which align with standardized, validated, international measures to allow greater comparison between populations.^6,10^

Recommendations for cleft services arising from the study are outlined below.

### Key Recommendations to Support Transition to Adulthood

1. The application of international standards to cleft care:
  Increased collaboration and communication between Cleft Teams, support groups, and University research teams for more unified and consistent services. Determining how to provide equitable services to all patients is essential. At a minimum, countries should develop a nationwide cleft service specification incorporating both clinical and lived experience perspectives and ratified by an appropriate government authority (eg, the Ministry of Health in New Zealand).
2. Routine implementation of Cleft Team Clinical Psychologists into each Cleft Team, alongside formal standardized psychological screening and monitoring protocols to identify patients experiencing psychological distress, and to ensure they are provided with the appropriate psychological support and follow-up as clinically indicated.^6,7,10,19^
3. Development of community-led supports including peer-support services pairing young adults with cleft, with adolescent patients who have recently transitioned or are soon to transition.^10,11,16^ Such support could be offered in person and/or online following consultation with stakeholders.^7,16^ Community-led supports would also offer opportunities for clinician-patient interactions and relationship-building outside the hospital setting,^3,18^ potentially reducing patients’ anxieties regarding their clinical experiences and decision-making.^10^

### Implications of Findings for Future Practice and Research

This study of Cleft Team clinician perspectives of transition has determined several considerations to improve patients’ experiences of cleft care in locations where formal transition pathways do not yet exist. Additionally, this study has revealed that further investigation for patients and their families is essential. Participants in this study frequently mentioned that their perspectives and recommendations may not fully correlate to those of patients and their families, a phenomenon that has also been described elsewhere.^5,21^ Furthermore, patients and their parents often report varying levels of psychosocial adjustment to having a cleft^26^ and satisfaction with patients’ appearance and perceived speech problems.^17^ There is a general paucity of research into patients’ perspectives and experiences of transition within cleft care internationally.^3^ However, a recent UK study does provide insight into patients’ perspectives of transition, revealing that patients encounter difficulties making treatment decisions independently and taking responsibility for their own care.^21^ These findings align with similar reports from clinicians within our study. To deepen our understanding of optimal cleft care, future studies to understand patients’ perspectives of transition are paramount, and incorporating patient voice into any proposed interventions is essential. Conducting international studies of patient perspectives of transition to adulthood similar to the one conducted in the United Kingdom^13^ is recommended to determine necessary considerations specific to each country's service provision and cultural context. Research investigating suitable social supports and coping strategies for families during transition to adulthood is also recommended,^27^ as family members of patients with cleft are impacted by patients’ cleft-related experiences.^5,10,17^

### Strengths and Limitations

The findings of this study present a novel perspective regarding the treatment experiences of patients in New Zealand with a cleft, and their families, as told from Cleft Team clinicians’ perspectives. The study incorporated a variety of perspectives from many different disciplines within cleft teams, and all-bar-one of the New Zealand cleft teams contributed to this study allowing for excellent geographic representation.

An acknowledged limitation of this study is that it did not seek patient perspectives of transition, however, consultation with patient representatives is a key stage of implementing the suggested actions of this study. Additionally, the research team contained a researcher with lived experience of cleft who offered a lived experience perspective to the design and analysis of this study.

## Conclusions

To facilitate smooth patient transition from pediatric to adult cleft care, the provision of holistic, lifelong care to patients and their families is necessary. The psychological well-being of patients and their families should be supported with regular psychological screening and monitoring and routine inclusion of Cleft Team Clinical Psychologists. Peer support services connecting adolescents and young adults who have experiences of cleft care as they transition from pediatric services is also recommended.
